# Multi‐institutional study on the commissioning and clinical implementation of the TrueBeam enhanced leaf model in the Eclipse treatment planning system

**DOI:** 10.1002/acm2.70360

**Published:** 2025-11-25

**Authors:** Ryohei Miyasaka, Ryuta Hirai, Yuhi Suda, Ryohei Yamauchi, Norifumi Mizuno, Yuya Suzuki, Mitsunobu Igari, Tomohiro Ohta, Hiroki Nakayama, Yu Arai, Kaito Sakai, Kazushi Hatakeyama, Hisayuki Miyashita, Masahiko Kurooka, Toru Kawachi, Ryusuke Hara

**Affiliations:** ^1^ Division of Radiation Oncology Chiba Cancer Center Chiba Chiba Japan; ^2^ Department of Radiation Oncology Saitama Medical University International Medical Center Hidaka Saitama Japan; ^3^ Department of Radiation Oncology Tokyo Metropolitan Cancer and Infectious Diseases Center Komagome Hospital Bunkyo‐ku Tokyo Japan; ^4^ Department of Radiation Oncology St. Luke's International Hospital Chuo‐ku Tokyo Japan; ^5^ Department of Radiation Oncology Saitama Medical Center Saitama Medical University Kawagoe Saitama Japan; ^6^ Department of Radiological Technology Tokyo Saiseikai Central Hospital Minato‐ku Tokyo Japan; ^7^ Radiation Safety and Quality Assurance Division National Cancer Center Hospital Chuo‐ku Tokyo Japan; ^8^ Department of Radiology Tokyo Metropolitan Tama Medical Center Fuchu Tokyo Japan; ^9^ Department of Radiation Control Radiological Oncology St. Marianna University Hospital Kawasaki Kanagawa Japan; ^10^ Department of Radiation Therapy Tokyo Medical University Hospital Shinjuku‐ku Tokyo Japan

**Keywords:** dose verification, ELM, MLC, multi‐institutional study, VMAT

## Abstract

**Purpose:**

Recently, the Enhanced Leaf Modeling (ELM) was developed to improve the multileaf collimator (MLC) model in the Eclipse treatment planning system (TPS). This multi‐institutional study evaluated the dose calculation accuracy and clinical feasibility of the ELM for TrueBeam linear accelerators (linacs) equipped with Millennium 120 and High‐Definition 120 MLCs. In addition, to facilitate broader clinical adoption, the feasibility of using machine‐averaged ELMs (ELM_Ave_) for both MLC types was evaluated.

**Methods:**

Twelve TrueBeam linacs from nine institutions were included. Machine‐specific ELM (ELM_MS_) parameters, including Leaf Transmission (LT) and the Leaf Gap (LG), were derived from vendor MLC sequences. Three volumetric modulated arc therapy (VMAT) plans for prostate cancer, head and neck cancer, and spinal metastasis were calculated using Acuros XB with each ELM_MS_ and verified using an ionization chamber and either Delta4 or ArcCHECK systems, based on relative dose differences and gamma pass rates (3%/2 mm). Verification criteria were set as tolerance and action levels of ≤2% and ≤3% for relative dose difference and ≥95% and ≥90% for gamma pass rate. To further improve the ELM dose calculation accuracy for VMAT cases, the LT and LG parameters were systematically adjusted based on the verification results and then averaged across all linacs to obtain the ELM_Ave,_ which was subsequently verified using the same procedure.

**Results:**

For the ELM_MS_ dose calculations, the relative dose differences across the three VMAT cases averaged −1.13%, with a two‐standard deviation (2 SD) of 1.04%. The mean gamma pass rate for all dose profiles was 99.4% (2 SD = 2.6%). The ELM_MS_ achieved clinically acceptable accuracy, with 96.3% of the verification results within clinical tolerance and no cases exceeding the action level. After parameter tuning, the ELM_Ave_ calculations exhibited dose differences within ±2% and gamma pass rates above 96% across all verification cases investigated in this study.

**Conclusions:**

The ELM_MS_ demonstrated reliable VMAT dose calculation accuracy across multiple institutions and MLC types. Tuning the LT and LG parameters can further improve ELM performance in various VMAT plans. The tuned ELM_Ave_ satisfied all clinical tolerance criteria in all verification cases and may serve as a practical approach for efficient commissioning and objective evaluation of ELM parameters across institutions.

## 1 INTRODUCTION

Advanced techniques such as intensity‐modulated radiation therapy and volumetric modulated arc therapy (VMAT) have significantly improved the spatial accuracy and conformity of dose delivery. Multileaf collimators (MLCs) are an integral component of these techniques, dynamically shaping the radiation fields and modulating photon fluence to ensure dose conformity within the target volume while sparing normal tissues. The sequence of MLC motions is optimized within the treatment planning system (TPS); thus, a detailed leaf model is required for accurate dose calculations.

In the Eclipse TPS (Varian Medical Systems, Palo Alto, CA, USA), the conventional leaf model (CLM) reproduces dose leakage characteristics using two primary parameters: Leaf Transmission (LT) and the Dosimetric Leaf Gap (DLG). The LT parameter is defined as the average of intra‐leaf and inter‐leaf transmission and is assumed to be constant across the entire leaf structure. The DLG parameter accounts for the obvious increase in the field size resulting from radiation transmitted through the rounded leaf tips, thereby reducing dose calculation uncertainties that may arise from simplified MLC models with straight leaf ends. Although these parameters provide an efficient and practical means of representing MLC characteristics, some studies have reported variable accuracy of the CLM in specific scenarios, such as VMAT.^1^, ^2^, ^3^, ^4^, ^5^, ^6^

Recently, an Enhanced Leaf Modeling (ELM) was developed to refine the MLC model in the Eclipse TPS. The ELM was developed via ray tracing of the actual leaf design, incorporating features such as the rounded tip shape of MLC leaves, the drive screw cutout, and the thickness of the leaf body.^7^ The ELM is designed for three MLC types manufactured by Varian Medical Systems: Millennium 120 (M120), High‐Definition 120 (HD120), and Halcyon SX. The ELM can be used for dose calculations with the AAA, Acuros XB, and Photon Optimizer (PO) algorithms (Varian Medical Systems, Palo Alto, CA, USA) in Eclipse version 18.0 and later. When the ELM is used in dose calculation processes, the uncertainty arising from a simplified MLC model can be reduced because it considers changes in photon fluence due to the path length inside the leaf body and the effect of the rounded leaf tip on the penumbra shape. Therefore, the ELM accounts for variations in LT observed across different leaf widths within the MLC bank, such as the 5 and 10 mm leaves in the M120 MLC and the 2.5 and 5 mm leaves in the HD120 MLC, which also vary depending on the beam energy. In addition, the Leaf Gap (LG) parameter reflects mechanical calibration and excludes the dose leakage effects caused by the rounded leaf tip, which are conventionally incorporated in the conventional DLG, thereby allowing the LG to adopt negative values. In the ELM, the LT and LG parameters are automatically configured in the Eclipse TPS by registering ionization chamber readings obtained using a vendor‐specified MLC sequence under reference conditions of a 10 cm depth in water and a source‐to‐surface distance (SSD) of 90 cm.

Van Esch et al. demonstrated that the HD120 and Halcyon SX ELMs provide more accurate dose predictions than the CLM in MLC‐defined slit fields using 6 MV x‐ray beams.^8^ This improvement was confirmed through experimental dose verification using various test plans, including static and dynamic MLC sequences. Moktan et al. reported that the dose calculations with the HD120 ELM were more correlated with measured dose distributions than the calculations obtained using the DLG‐based MLC model for single‐isocenter multitarget (SIMT) VMAT plans using 6‐MV flattening‐filter‐free (FFF) beams.^9^ Their findings demonstrated that the ELM enables improved dose calculation accuracy for small dynamic MLC apertures at both the central and off‐axis positions. Miyasaka et al. confirmed that incorporating the Halcyon SX ELM into the dose calculation process significantly improved the accuracy of VMAT plan verifications.^10^ They concluded that the observed improvement resulted from the greater ability of the ELM to simulate the actual leaf‐tip transmission of the dual‐layer MLC, both with and without leaf trailing.

Although previous studies on the ELM have demonstrated improvements in dose calculation accuracy and supported its feasibility for clinical implementation, they have not included data for the M120 ELM and have been limited to 6 MV beams, even for other ELM types. In addition, clinical validation of the HD120 ELM has been limited to SIMT VMAT plans, with no commissioning performed for standard clinical sites, such as the pelvis or head and neck regions. All previous studies are also limited to single‐institution investigations. Therefore, a multi‐institutional study is essential for a comprehensive evaluation of the feasibility of clinical implementation of the ELM. The purpose of this study is to comprehensively evaluate the accuracy of the ELM, particularly for the M120 and HD120 MLCs, across various treatment cases and beam energies through a multi‐institutional study. Furthermore, to facilitate broader and more efficient clinical adoption of the ELM across diverse institutions, this study assessed the feasibility of implementing machine‐averaged ELMs for both MLC types.

## 2 MATERIALS AND METHODS

As summarized in Table 1, this study investigated 12 TrueBeam linear accelerators (linacs) installed across nine clinical facilities. Each TrueBeam linac was equipped with either an M120 or HD120 MLC, and x‐ray beams with either flattened or FFF modes were available at photon energies of 6 and/or 10 MV. Routine quality assurance (QA) procedures for each TrueBeam system were systematically performed at each clinical facility in accordance with the recommendations outlined in the American Association of Physicists in Medicine (AAPM) Task Group reports.^11^, ^12^

**TABLE 1:** Details of TrueBeam linear accelerator at each clinical site.

TrueBeam #	Clinical site	MLC type	X‐ray beam energy
1	A	M120	6 MV, 6 MV‐FFF
2	B	M120	6 MV, 6 MV‐FFF, 10 MV
3	C	M120	6 MV, 6 MV‐FFF, 10 MV, 10 MV‐FFF
4	D	M120	6 MV, 6 MV‐FFF, 10 MV, 10 MV‐FFF
5	E	M120	6 MV, 6 MV‐FFF, 10 MV, 10 MV‐FFF
6	F	M120	6 MV, 6 MV‐FFF, 10 MV, 10 MV‐FFF
7	G	M120	6 MV, 6 MV‐FFF, 10 MV, 10 MV‐FFF
8	H	M120	6 MV, 6 MV‐FFF, 10 MV, 10 MV‐FFF
9	B	HD120	6 MV, 6 MV‐FFF, 10 MV, 10 MV‐FFF
10	C	HD120	6 MV, 6 MV‐FFF, 10 MV, 10 MV‐FFF
11	D	HD120	6 MV, 6 MV‐FFF, 10 MV, 10 MV‐FFF
12	I	HD120	6 MV, 6 MV‐FFF, 10 MV, 10 MV‐FFF

In this study, all treatment plans were developed in the Eclipse research environment (version 18.0) and calculated using the Acuros XB dose calculation algorithm. The VMAT plans were optimized using the PO algorithm (version 18.0). For dose calculations, a grid size of 2 mm was used for the open fields and the standard VMAT plans, whereas a finer grid size of 1 mm was employed for the narrow‐slit fields and the stereotactic VMAT plans. Throughout the study, data processing and analysis were performed using an in‐house program (MATLAB R2021a, Mathworks, Natick, MA, USA).

### 2.1 Quality assurance for VMAT dose delivery

The variability in machine performance during VMAT dose delivery for each TrueBeam linac was quantified using output measurements and log file analysis. As detailed in Table 2, VMAT plans using 1–3 arcs were generated for nonclinical patient cases,^13^ each corresponding to a specific combination of MLC type and x‐ray beam energy.

**TABLE 2:** The summary of plan characteristics for each volumetric modulated arc therapy (VMAT).

Case #	Target	Prescribed dose (Gy/fraction)	Target volume (cc)	Arc geometry	Treatment strategy
1	Prostate cancer	3.0	127.7	1 Full arc	Standard VMAT
2	Head and neck cancer	2.0, 1.8 or 1.6	596.6	3 Full arcs	Simultaneous integrated boost
3	Spinal metastases	7.0	38.2	2 Full arcs	Stereotactic VMAT

VMAT output was measured using a water‐equivalent cubic phantom (Tough Water, Kyoto Kagaku, Japan) containing an inserted ionization chamber (31021, PTW, Freiburg, Germany). For each case, the ionization chamber was placed at the center of the target volume, and the point doses were measured. The chamber position corresponded to the isocenter for Cases #1 and #3 and to an off‐isocenter position for Case #2. The output consistency among the TrueBeam linacs was evaluated using the coefficient of variation (CV) of the measured doses, calculated as “(standard deviation [SD]/mean) × 100.”

Log file analysis was performed to evaluate the mechanical reproducibility of leaf positions, monitor units (MU), and gantry rotation angles during VMAT irradiation. Mechanical reproducibility was quantified as the absolute difference between the planned and recorded values during each control cycle. To evaluate the machine performance accuracy across the three VMAT cases, the root mean square (RMS) and 95th percentile of delivery errors were calculated over the control cycles.

### 2.2 Beam model configuration in Eclipse TPS

The beam models for all TrueBeam linacs were configured in the Eclipse TPS using Acuros XB (version 18.0). The reference dose for each beam model, which was defined as the absorbed dose at an SSD of 90 cm and a depth of 10 cm in water, using a 10 cm × 10 cm field with 100 MU irradiation, was determined based on calibration values specific to each TrueBeam linac. The effective target spot sizes were set to the default values specified by Acuros XB version 18.0, namely 0.5 mm in the *X*‐direction and 0.7 mm in the *Y*‐direction. All other open‐beam parameters were configured using the representative beam data (RBD) for TrueBeam.^14^ The open‐beam model was verified by comparison with measurements from each linac. The reference dose was evaluated using the relative dose difference, which was calculated using the formula “(*D*_calc_ − *D*_meas_)/*D*_meas_ × 100,” where *D*_calc_ denotes the calculated dose and *D*_meas_ denotes the measured dose. The depth and lateral dose profiles were assessed using gamma pass rates at field sizes of 3 cm × 3 cm, 10 cm × 10 cm, and 30 cm × 30 cm. Gamma analysis was performed using criteria of a dose difference of 3% and distance‐to‐agreement (DTA) of 2 mm, with a lower dose threshold of 10% (3%/2 mm, 10%).

The dosimetric characteristics of the MLC were reproduced by the ELM using the Acuros XB algorithm. To generate machine‐specific ELMs (ELM_MS_) for the 12 TrueBeam linacs, measured charge values were obtained for open, leaf‐shielded, and sweeping gap fields with gap widths of 4, 6, and 20 mm, respectively, all within a 10 cm × 10 cm collimated field, following the MLC sequences provided by the vendor. All measurements were performed using a Farmer‐type ionization chamber positioned at the isocenter within a water phantom under reference conditions. The measured charge values were imported into the Eclipse TPS, and the LT and LG parameters were subsequently calculated.

### 2.3 Testing of machine‐specific ELM

To evaluate the dose calculation accuracy of the ELM_MS_ under dynamic MLC conditions, a sweeping gap test and dose verification of the VMAT treatment plans were performed.

**FIGURE 1:**
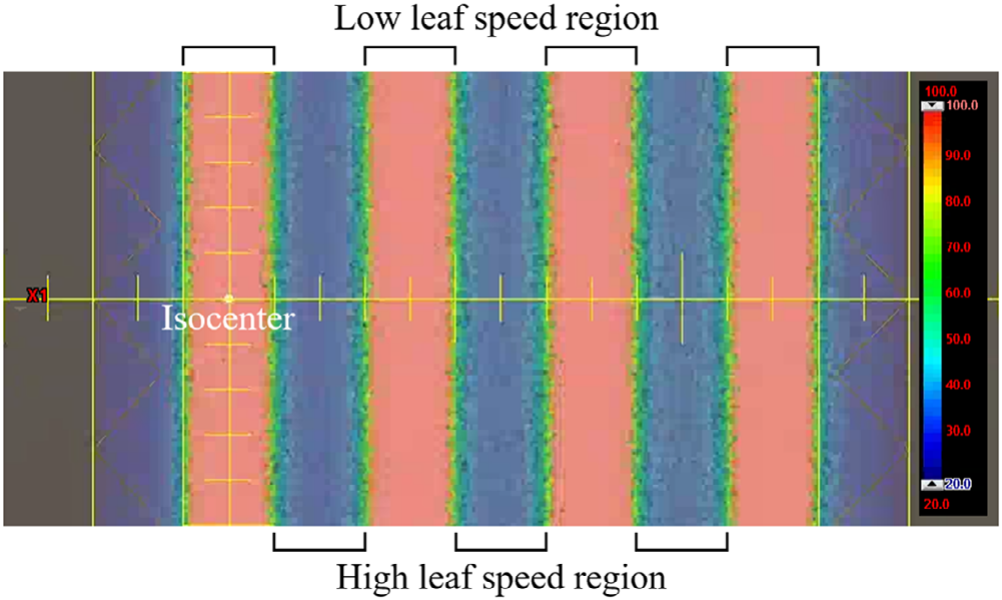
Beam's eye view of the calculated striped dose distribution using the Dynamic Zebra Crosswalk field. The delivered dose increases in regions with low leaf speeds and decreases in those with high leaf speeds.

In the sweeping gap test, a narrow gap width of 0.4 cm was used, and leaf speeds were modulated to vary the photon fluence, resulting in striped dose distributions with a 2 cm period along the central beam axis as well as at off‐axis positions 4, 8, and 12 cm from the isocenter, as shown in Figure 1. This methodological approach was originally proposed by Van Esch et al. as the Dynamic Zebra Crosswalk (DZC) test and has been proven to be a reliable technique for evaluating the modeling accuracy of MLC parameters.^8^ The dose profiles of the DZC fields were measured using a radiochromic film (Gafchromic EBT4, Ashland Inc., USA) placed at a depth of 10 cm in the Toughwater phantom. The calculated DZC profiles were obtained using ELM_MS_ and subsequently validated against the film measurements using a two‐dimensional global gamma index (3%/2 mm, 10%) in QA software (Radiological Imaging Technology Inc., USA). Absolute dose differences between each measured and calculated point were normalized to the maximum measured dose, and the gamma pass rates were evaluated.

In the VMAT dose verification process, the three patient plans listed in Table 1 were recalculated using the ELM_MS_ within virtual phantoms simulating QA devices: the Tough Water cubic phantom with an inserted 31021 chamber and either the Delta4 system (ScandiDos Inc., Ashland, VA, USA) or the ArcCHECK system (Sun Nuclear Corporation, Melbourne, FL, USA). These plans were delivered to the corresponding QA devices on each TrueBeam linac. A consistent phantom and ionization chamber setup were used for point dose measurements at all facilities. The Delta4 system was used at clinical sites A, B, C, D, F, G, and H, and the ArcCHECK system was used at clinical sites E and I. Before all measurements, the dose per MU was established according to the standard dosimetry protocol, and daily output variations were corrected. For single‐point dose verification, the calculated doses were compared with the ionization chamber measurements, and the relative dose differences were evaluated. In addition, the calculated dose profiles were validated against the measurements obtained using the Delta4 or ArcCHECK systems operated in absolute dose mode using a three‐dimensional global gamma index (3%/2 mm, 10%). The corresponding gamma pass rates were also evaluated. VMAT dose verification criteria were defined according to the AAPM Task Group 218 report, with tolerance and action levels of ≤2% and ≤3% for the relative dose difference and ≥95% and ≥90% for the gamma pass rate, respectively.^15^

### 2.4 Adjustment of ELM parameters for VMAT dose calculation

To improve the dose calculation accuracy of the ELM_MS_, the LT and LG parameters were systematically adjusted based on both the DZC test and VMAT dose verification results. Because the LT parameter significantly impacts dose calculation accuracy in the valley regions of the DZC distribution, each measured LT value was individually adjusted to ensure close agreement between the calculated and measured dose profiles, as described in Section 2.3. This adjustment was validated by evaluating the gamma pass rates (3%/2 mm, 10%). The parameter sensitivities for LT are detailed in Table S1, and the adjusted values are hereafter referred to as LT_Tuned_.

The VMAT plans were recalculated within a virtual phantom using the combination of LT_Tuned_ and the measured LG for each TrueBeam linac. The recalculated single‐point doses were quantitatively compared with the ionization chamber measurements described in Section 2.3 by evaluating the mean relative dose differences. Previous studies have typically adjusted parameters related to the LG width, such as the DLG, to improve the accuracy of target dose calculations in VMAT.^1^, ^2^, ^3^, ^4^, ^5^, ^6^ Therefore, in this study, each LG parameter was slightly modified, based on the parameter sensitivities shown in Table S2, to achieve the best agreement between the calculated and measured point doses across the three VMAT cases. The LG value that minimized the mean relative dose difference was defined as the empirically adjusted LG (LG_Tuned_).

For each beam model, the ELM was refined into a tuned model (ELM_Tuned_), comprising the combined set of the LT_Tuned_ and LG_Tuned_ parameters. The VMAT plans were recalculated within virtual Delta4 or ArcCHECK phantoms using the ELM_Tuned_, and the dose profiles were validated against measurements described in Section 2.3 through the evaluation of the gamma pass rates (3%/2 mm, 10%).

### 2.5 Clinical feasibility of averaged ELMs

To facilitate broader and more efficient clinical adoption of the ELM across diverse institutions, this study investigated the feasibility of implementing machine‐averaged ELMs (ELM_Ave_) for M120 and HD120 MLC. The ELM_Ave_ for each MLC type was defined as the combination of the averaged LT_Tuned_ and LG_Tuned_ parameters specific to each configuration. The ELM_Ave_ was incorporated as an add‐on to the beam model for each TrueBeam linac. Subsequently, three VMAT plans were recalculated, and the resulting doses were evaluated against the measured point doses and dose distributions, as described in Section 2.3. The evaluation focused on relative dose differences and gamma index analysis (3%/2 mm, 10%).

## 3 RESULTS

### 3.1 Quality assurance for VMAT dose delivery

**FIGURE 2:**
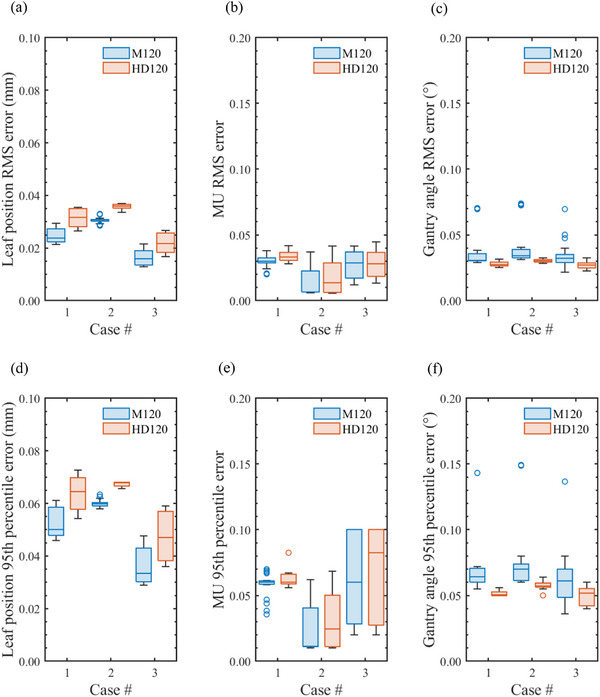
Boxplots of the root mean square (RMS) and 95th percentile errors during VMAT dose delivery in three patient cases using the Millennium 120 (M120) and High‐Definition 120 (HD120) MLCs. These errors were derived from log file analyses of (a, d) MLC leaf positions, (b, e) monitor units (MU), and (c, f) gantry rotation angles.

Figure 2 shows the RMS and 95th percentile of mechanical errors during VMAT dose delivery in three patient cases using the M120 and HD120 MLC types, derived from log file analyses of (a, d) MLC leaf positions, (b, e) MU, and (c, f) gantry rotation angles. Among all linacs, TrueBeam #2 tended to produce more gantry angle errors than the other linacs. However, these deviations were confirmed to be minimal and well within the tolerance limits recommended by AAPM Task Group reports.^11^, ^12^ Consequently, all linacs exhibited sufficiently high mechanical accuracy during VMAT delivery, irrespective of the case or MLC type. Table 3 summarizes the CV for the measured doses in each VMAT case. The VMAT irradiation performed on the TrueBeam linacs exhibited excellent output stability for both the M120 and HD120 MLCs, with maximum deviations not exceeding 0.6%.

**TABLE 3:** Coefficient of variation (CV) of the measured point dose in each VMAT case using either the Millennium 120 (M120) or High‐Definition 120 (HD120) MLCs.

	CV of measured point doses (%)	
Case #	M120 MLC	HD120 MLC
1	0.28	0.39
2	0.49	0.18
3	0.52	0.28

### 3.2 Beam model configuration in Eclipse TPS

Table 4 presents the mean, SD, and range of the relative dose differences under reference conditions for each x‐ray beam. The maximum calculation error was 0.24%, which was slightly more pronounced for the 6 MV x‐ray beam; however, all reference doses remained within clinically acceptable limits.

**TABLE 4:** Summary statistics (mean, standard deviation (SD), and range) of the relative dose differences under reference conditions, categorized by x‐ray beam energy.

	Relative dose difference under reference condition (%)			
	6X	6X‐FFF	10X	10X‐FFF
Mean	−0.22	−0.22	−0.16	−0.03
SD	0.02	0.01	0.02	0.01
Max	−0.17	−0.22	−0.12	−0.01
Min	−0.24	−0.24	−0.18	−0.06

**FIGURE 3:**
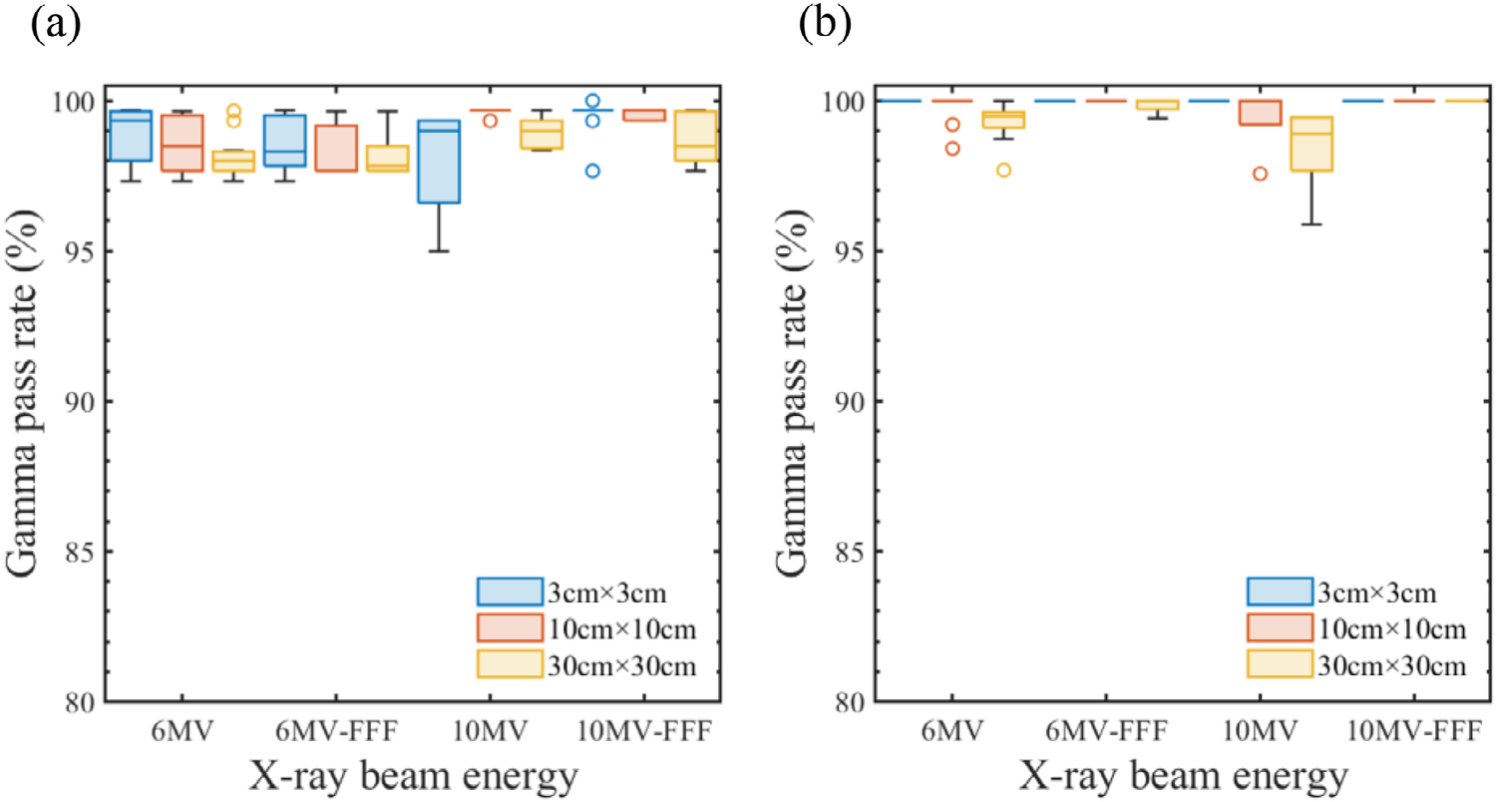
Boxplots of the gamma pass rates (3%/2 mm, 10%) for the (a) depth and (b) lateral dose profiles at field sizes of 3 cm × 3 cm, 10 cm × 10 cm, and 30 cm × 30 cm.

Figure 3 shows box plots of the gamma pass rates (3%/2 mm, 10%) for the (a) depth and (b) lateral dose profiles at field sizes of 3 cm × 3 cm, 10 cm × 10 cm, and 30 cm × 30 cm. These plots present a comparison between open‐beam model calculations and linac‐specific measurements, categorized by the x‐ray beam energy. Across x all dose profiles, the mean gamma pass rate was 99.2% (SD = 1.0%). No facility reported a pass rate below 95%.

Tables 5 and 6 list the LT and LG parameters in the ELM_MS_ for the TrueBeam linacs equipped with M120 or HD120 MLCs. For each MLC type, the SD of LT was below 0.001, indicating minimal inter‐machine variability. The LG exhibited an SD of approximately 0.016 cm, with a maximum difference between linacs of less than 0.050 cm. These results suggest that the leaf positions were calibrated with high precision for both M120 and HD120 MLCs.

**TABLE 5:** The Leaf Transmission (LT) and Leaf Gap (LG) parameter values in the machine‐specific ELMs (ELM_MS_) for TrueBeam equipped with the Millennium 120 multileaf collimator.

	LT				LG (cm)			
TrueBeam #	6X	6X‐FFF	10X	10X‐FFF	6X	6X‐FFF	10X	10X‐FFF
1	0.0145	0.0124	–	–	− 0.0664	− 0.0732	–	–
2	0.0146	0.0123	0.0169	–	− 0.0692	− 0.0764	− 0.0602	–
3	0.0148	0.0126	0.0170	0.0152	− 0.0300	− 0.0384	− 0.0191	− 0.0238
4	0.0146	0.0123	0.0167	0.0149	− 0.0439	− 0.0512	− 0.0364	− 0.0402
5	0.0141	0.0120	0.0164	0.0148	− 0.0364	− 0.0433	− 0.0286	− 0.0347
6	0.0143	0.0121	0.0164	0.0147	− 0.0262	− 0.0314	− 0.0175	− 0.0214
7	0.0131	0.0111	0.0151	0.0135	− 0.0329	− 0.0392	− 0.0246	− 0.0274
8	0.0140	0.0118	0.0162	0.0145	− 0.0447	− 0.0534	− 0.0384	− 0.0410
Mean	0.0143	0.0121	0.0164	0.0146	− 0.0437	− 0.0508	− 0.0321	− 0.0314
SD	0.0005	0.0005	0.0006	0.0006	0.0162	0.0164	0.0147	0.0084

**TABLE 6:** The Leaf Transmission (LT) and Leaf Gap (LG) parameter values in the machine‐specific ELMs (ELM_MS_) for TrueBeam equipped with the High‐Definition 120 multileaf collimator.

	LT				LG (cm)			
TrueBeam #	6X	6X‐FFF	10X	10X‐FFF	6X	6X‐FFF	10X	10X‐FFF
9	0.0111	0.0094	0.0127	0.0114	− 0.0252	− 0.0303	− 0.0213	− 0.0239
10	0.0119	0.0102	0.0136	0.0121	− 0.0051	− 0.0104	− 0.0001	− 0.0014
11	0.0118	0.0100	0.0136	0.0122	− 0.0274	− 0.0314	− 0.0223	− 0.0251
12	0.0121	0.0102	0.0138	0.0124	− 0.0334	− 0.0369	− 0.0285	− 0.0300
Mean	0.0117	0.0100	0.0134	0.0120	− 0.0228	− 0.0273	− 0.0180	− 0.0201
SD	0.0004	0.0004	0.0005	0.0004	0.0123	0.0116	0.0124	0.0127

### 3.3 Testing of machine‐specific ELM

**FIGURE 4:**
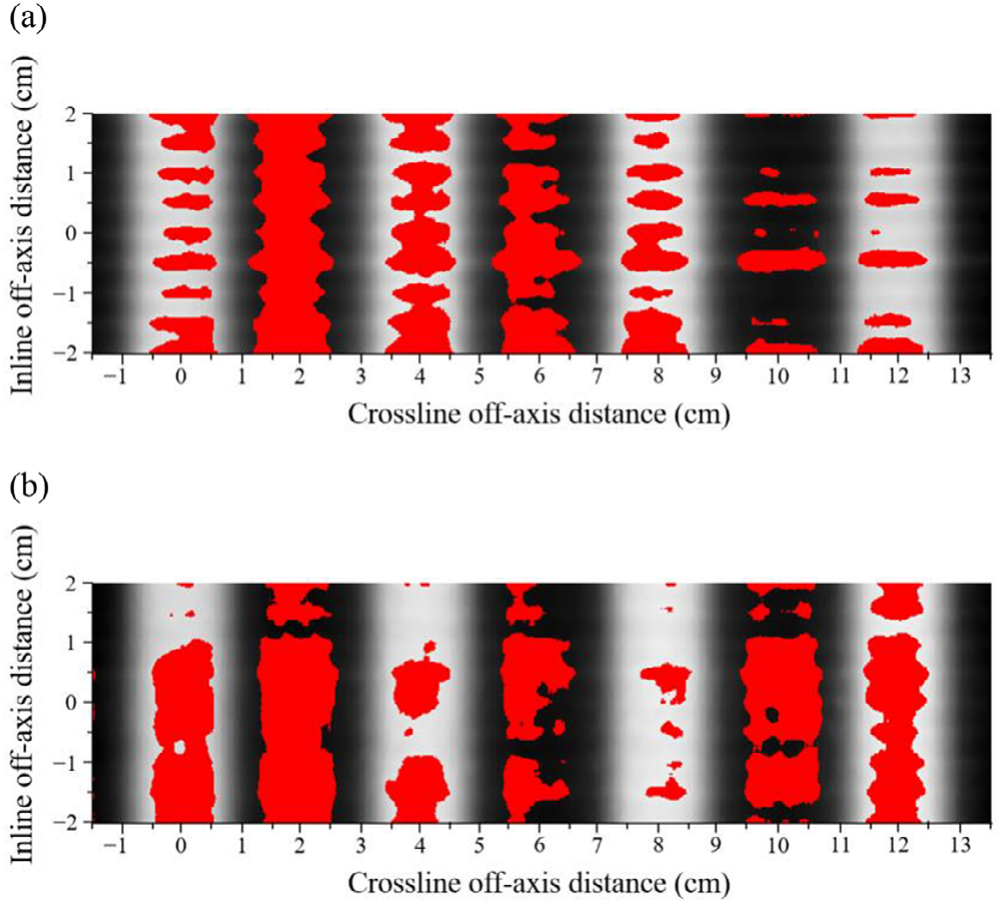
Measured dose distributions obtained from the Dynamic Zebra Crosswalk test conducted using either (a) the Millennium 120 or (b) the High‐Definition 120 MLCs. Regions where the gamma index (3%/2 mm, 10%) of the calculated dose exceeds 1 are highlighted in red.

Figure 4 shows a representative measured dose distribution obtained from the DZC test conducted using either (a) M120 or (b) HD120 MLCs. Regions in which the gamma index of the calculated dose exceeds 1, indicating a dose difference > 3% or DTA > 2 mm, are highlighted by red markers. When the ELM_MS_ was incorporated into the beam model, the calculated dose profiles tended to underestimate the dose relative to the film measurements, particularly in the valley regions of the DZC distribution, where the dose discrepancy exceeded 10%. Table 7 summarizes the mean and SD of the gamma pass rates (3%/2 mm, 10%) for those distributions using the ELM_MS_ with the M120 and HD120 MLCs; overall pass rates were low, and only a few cases exceeded 90%. The gamma analysis failures observed in the valley regions can predominantly be ascribed to an underestimation of the LT parameter in the ELM_MS_.

**TABLE 7:** Mean and standard deviation (SD) of the gamma pass rates (3%/2 mm, 10%) comparing the dose calculations using machine‐specific ELMs (ELM_MS_) and film measurements in the Dynamic Zebra Crosswalk test conducted with either the Millennium 120 (M120) or High‐Definition 120 (HD120) MLCs.

	Gamma pass rate (%)							
	M120 MLC				HD120 MLC			
	6X	6X‐FFF	10X	10X‐FFF	6X	6X‐FFF	10X	10X‐FFF
Mean	76.3	72.2	74.4	75.4	72.7	73.0	76.5	78.7
SD	10.9	9.8	7.9	13.5	9.0	8.3	8.1	11.3

**FIGURE 5:**
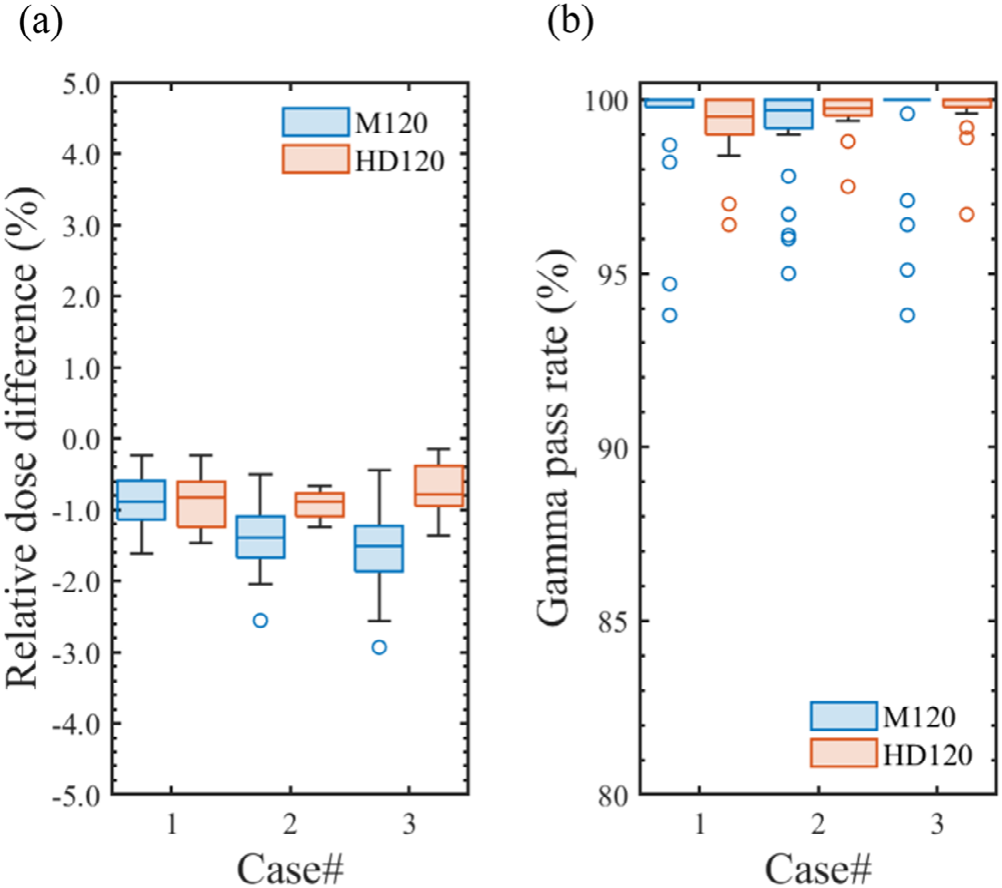
Boxplots of (a) the relative dose differences and (b) the gamma pass rates (3%/2 mm, 10%) between the calculated and measured doses for three VMAT cases, using machine‐specific ELMs (ELM_MS_) for either the Millennium 120 (M120) or High‐Definition 120 (HD120) MLCs.

In the VMAT dose verification, the relative dose differences across the three cases averaged −1.13%, with a 2 SD of 1.04%, and the mean gamma pass rate for all dose profiles was 99.4% (2 SD = 2.6%). Figure 5 shows box plots of the (a) relative dose differences and (b) the gamma pass rates (3%/2 mm, 10%) for TrueBeam linacs equipped with either the M120 or HD120 MLCs, based on each case. For the M120 ELM_MS_, the mean and 2 SD of the relative dose differences were −0.87% and 0.71% for Case #1, −1.42% and 0.85% for Case #2, and −1.57% and 1.13% for Case #3, respectively. In the case of the HD120 ELM_MS_, these values were −0.91% and 0.76%, −0.93% and 0.37%, and −0.74% and 0.77% for Cases #1, #2, and #3, respectively. The verification results for each x‐ray energy are summarized in Tables S3 and S4, presenting relative dose differences and gamma pass rates, respectively. Based on the dose calculations obtained using the M120 ELM, although Cases #2 and #3 exhibited greater dose deviations than Case #1, the maximum difference was within the action level. Regardless of the MLC type, most calculated doses achieved gamma pass rates above 95%. However, several institutions showed reduced pass rates in Case #2, and the gamma analysis performed using ArcCHECK for Cases #1 and #3 occasionally failed to meet the tolerance level.

### 3.4 Adjustment of ELM parameters for VMAT dose calculation

**FIGURE 6:**
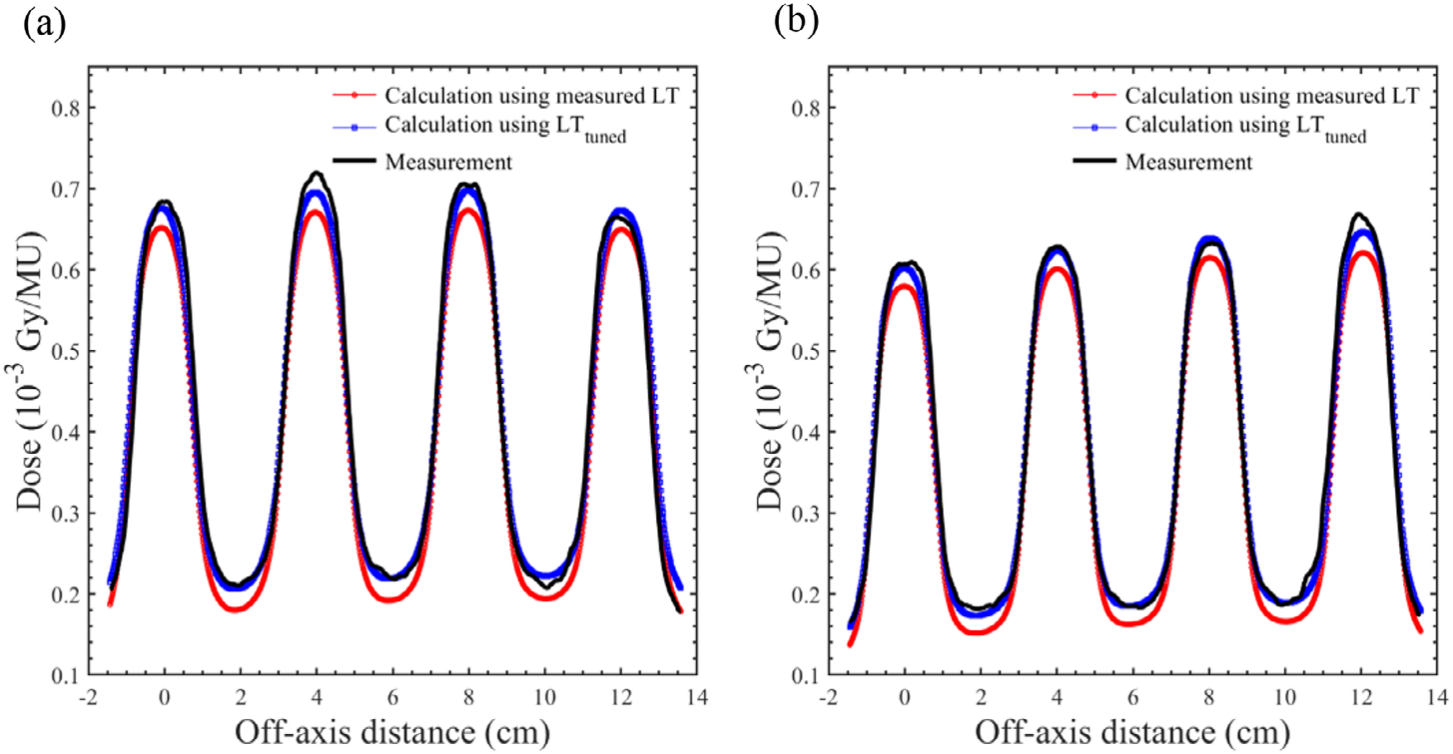
The calculated and measured Dynamic Zebra Crosswalk dose distributions for a 6 MV x‐ray beam with (a) the Millennium 120 and (b) the High‐Definition 120 MLCs. The calculations were performed based on the measured LT and LT_Tuned_ values, and the measurements were obtained using film.

To ensure close agreement between the calculated DZC distributions and film measurements, the LT_Tuned_ values were determined as detailed in Table 8. Figure 6a shows the DZC dose distributions calculated using the measured LT and LT_Tuned_ values for a 6 MV beam with an M120 MLC, and Figure 6b shows the corresponding distributions for a 6 MV beam with an HD120 MLC. The introduction of LT_Tuned_ resulted in a significant enhancement in the valley doses and a slight increase in the peak doses, resulting in a substantial improvement in the agreement between the calculated and measured DZC distributions. Table 9 summarizes the mean and SD of the gamma pass rates (3%/2 mm, 10%) for the DZC distributions calculated using LT_Tuned_ for both the M120 and HD120 MLCs. All beam types exhibited average gamma pass rates above 90%, indicating good agreement between the calculated and measured dose distributions.

**TABLE 8:** Mean and standard deviation (SD) of the tuned Leaf Transmission (LT_Tuned_) parameter values for the Millennium 120 (M120) or High‐Definition 120 (HD120) MLCs.

	LT_Tuned_							
	M120 MLC				HD120 MLC			
	6X	6X‐FFF	10X	10X‐FFF	6X	6X‐FFF	10X	10X‐FFF
**Mean**	**0.0188**	**0.0166**	**0.0227**	**0.0199**	**0.0151**	**0.0137**	**0.0181**	**0.0165**
SD	0.0016	0.0021	0.0015	0.0028	0.0013	0.0016	0.0015	0.0021

**TABLE 9:** Mean and standard deviation (SD) of the gamma pass rates (3%/2 mm, 10%) comparing dose calculations using LT_Tuned_ with film measurements in the Dynamic Zebra Crosswalk distribution for Millennium 120 (M120) and the High‐Definition 120 (HD120) MLCs.

	Gamma pass rate (%)							
	M120 MLC				HD120 MLC			
	6X	6X‐FFF	10X	10X‐FFF	6X	6X‐FFF	10X	10X‐FFF
Mean	95.0	94.2	92.3	97.6	96.4	94.6	93.8	97.4
SD	1.6	2.7	3.0	1.2	2.5	3.3	3.9	2.3

To achieve optimal agreement between the calculated and measured point doses for the three VMAT plans, the LG_Tuned_ values were determined as detailed in Table 10. During M120 ELM tuning, LG_Tuned_ was determined such that the mean relative dose differences were 0.11% ± 0.55%, −0.22% ± 0.71%, and 0.13% ± 0.72% for Cases #1, #2, and #3, respectively, where ± indicates a range of 2 SD. For the HD120 ELMs, the corresponding mean and 2 SD values were −0.24% ± 0.59%, −0.12% ± 0.42%, and 0.40% ± 0.69% for Cases #1, #2, and #3, respectively.

**TABLE 10:** Mean and standard deviation (SD) of the tuned Leaf Gap (LG_Tuned_) parameter values derived from VMAT point dose verification results for the Millennium 120 (M120) or High‐Definition 120 (HD120) MLCs.

	LG_Tuned_ (cm)							
	M120 MLC				HD120 MLC			
	6X	6X‐FFF	10X	10X‐FFF	6X	6X‐FFF	10X	10X‐FFF
**Mean**	**−0.0307**	**−0.0289**	**−0.0273**	**−0.0051**	**−0.0127**	**−0.0131**	**−0.0137**	**−0.0014**
SD	0.0162	0.0159	0.0129	0.0184	0.0101	0.0142	0.0147	0.0246

**FIGURE 7:**
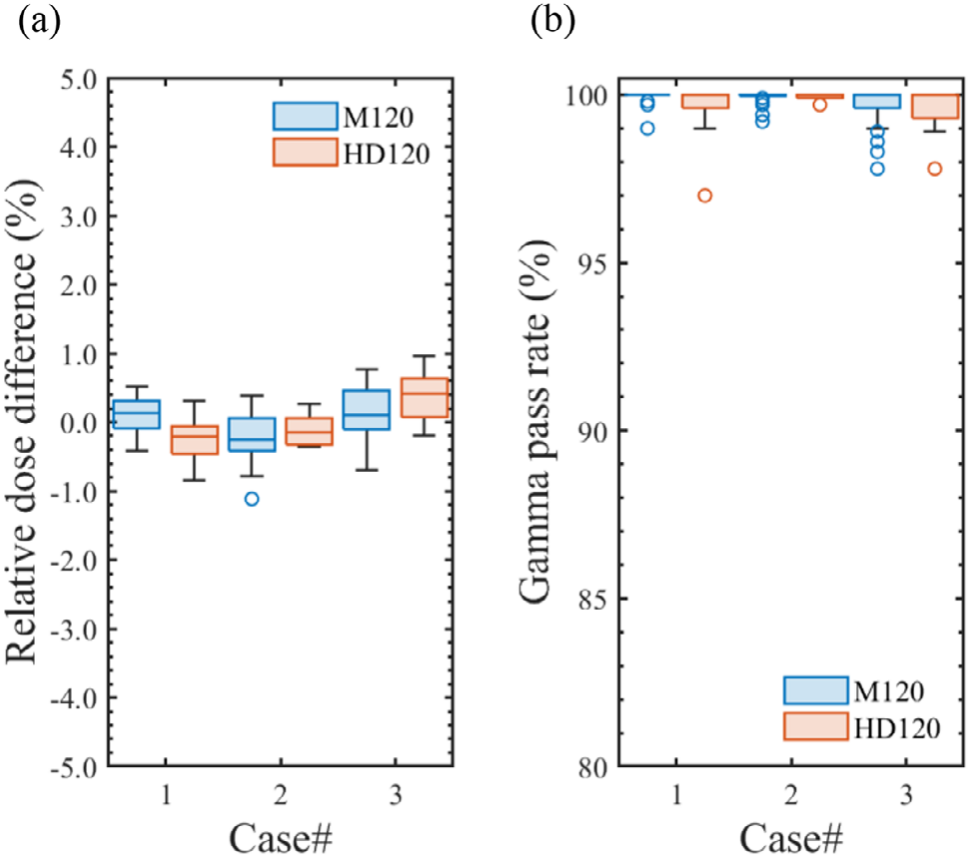
Boxplots of (a) the relative dose differences and (b) the gamma pass rates (3%/2 mm, 10%) obtained from VMAT dose verifications following the application of ELM_Tuned_ values for either the Millennium 120 (M120) or High‐Definition 120 (HD120) MLCs.

The ELM_Tuned_ parameter values for each TrueBeam are summarized in Tables S5 and S6. Figure 7 shows case‐wise box plots of (a) relative dose difference and (b) gamma pass rates (3%/2 mm, 10%) obtained from VMAT dose verifications following the application of individually determined ELM_Tuned_ values for each beam model. The ELM_Tuned_ for the M120 MLC yielded high mean gamma pass rates of 99.9% (range: 99.0%–100%) for Case #1, 99.9% (range: 99.2%–100%) for Case #2, and 99.7% (range: 97.8%–100%) for Case #3. For the HD120 MLC, the corresponding mean gamma pass rates were 99.6% (range: 97.0%–100%) for Case #1, 99.9% (range: 99.7%–100%) for Case #2, and 99.6% (range: 97.8%–100%) for Case #3. The calculated dose distributions exhibited excellent agreement with the measurements, exceeding the 95% acceptance criterion for all verification cases across all TrueBeam linacs.

### 3.5 Clinical feasibility of averaged ELMs

**FIGURE 8:**
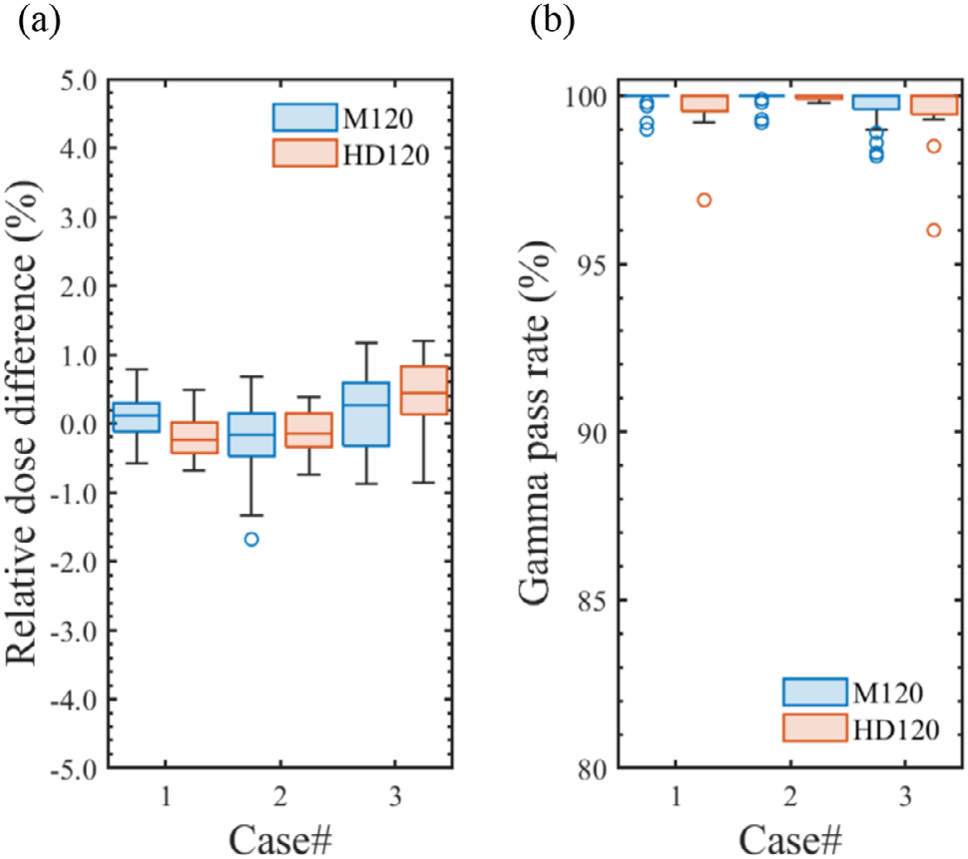
Boxplots of (a) relative dose differences and (b) gamma pass rates (3%/2 mm, 10%) from VMAT dose verifications using averaged ELM (ELM_Ave_) parameters for either the Millennium 120 (M120) or High‐Definition 120 (HD120) MLCs.

For the M120 and HD120 MLCs, the ELM_Ave_ for each beam type was defined as the average of the LT_Tuned_ and LG_Tuned_ values listed in Tables 8 and 10 and then applied to the corresponding beam model. In dose calculations using ELM_Ave_, the mean relative dose difference and gamma pass rate across the three VMAT cases were 0.02% (2 SD = 1.01%) and 99.8% (2 SD = 1.1%), respectively. All VMAT plans satisfied the tolerance levels for both the relative dose difference and gamma pass rate across each TrueBeam linac, regardless of the MLC type. Figure 8 shows case‐wise box plots of (a) the relative dose differences and (b) gamma pass rates (3%/2 mm, 10%) obtained from VMAT dose verifications after applying the ELM_Ave_ parameter sets for the M120 or HD120 MLCs. For the M120 ELM_Ave_, the mean and 2 SD of the relative dose differences were 0.11% and 0.70% for Case #1, −0.21% and 1.05% for Case #2, and 0.15% and 1.13% for Case #3, respectively. In the case of the HD120 ELM_Ave_, these values were −0.21% and 0.66%, −0.13% and 0.65%, and 0.38% and 1.19% for Cases #1, #2, and #3, respectively. For standard VMAT plans (e.g., Cases #1 and #2), as shown in Figures 5, 7, and 8, the ELM_Ave_ effectively resolved the discrepancies in dose distributions calculated with the ELM_MS_ parameter set while exhibiting only minor deviations from the ELM_Tuned_ results, which were individually adjusted for each linac. Slight discrepancies in the calculated dose were observed in stereotactic VMAT cases (e.g., Case #3) after applying the ELM_Ave_, which can be attributed to the absence of machine‐specific parameters. Although this concern may be clinically negligible, users should exercise caution and consider it carefully when implementing the ELM_Ave_ in clinical practice.

## 4 DISCUSSION

This study was conducted as a multi‐institutional investigation to evaluate the calculation accuracy of the ELM across multiple TrueBeam linacs. QA of these linacs was performed by log analysis and output measurements during VMAT dose delivery. Log analysis confirmed that the mechanical performance consistently remained within the recommended tolerance limits.^11^, ^12^ Nevertheless, although machine log data are extensively used to verify linac performance, they cannot detect calibration errors.^16^, ^17^ To comprehensively evaluate mechanical accuracy, the VMAT output was measured using an ionization chamber, and inter‐linac output variations were evaluated. The output variations among linacs were minimal for both the M120 and HD120 MLCs, with the maximum deviation not exceeding 0.6%. These results support the validity of the present study.

In this study, the beam model was developed using the RBD with the exception of the reference absolute dose. The reliability of the RBD has been extensively validated,^14^, ^18^, ^19^ and the calculation accuracy of the configured open‐beam model showed excellent agreement with the beam profiles of each linac. Previous studies have reported that differences between beam models constructed from the RBD and machine‐specific beam data can affect dose calculations in VMAT plans.^20^ These potential discrepancies represent a limitation of this study. The ELM_MS_ determined using vendor‐provided leaf sequences showed small variations for both the M120 and HD120 MLCs (Tables 5 and 6), confirming that each leaf position was calibrated to a high accuracy of less than 0.05 cm. The average parameters obtained in this study were in good agreement with those reported in previous research,^8^, ^9^ indicating that the MLC quality was maintained within internationally acceptable limits.^11^, ^12^ No previous reports have provided multi‐institutional ELM parameters for different MLC types; thus, these results can serve as a valuable reference for users implementing the ELM clinically.

The calculation accuracy of the ELM_MS_ was verified through DZC tests and VMAT dose verification. The calculated DZC fields exhibited discrepancies compared to the film measurements, with a pronounced tendency to underestimate the dose in the valley regions. In previous study, Van Esch et al. used high‐resolution detector arrays with limited measurement areas, which required dividing the DZC field into four separate stripes to validate the calculated doses.^8^ In contrast, this study used a single composite DZC field, potentially resulting in the MLC‐shielded regions being exposed for approximately four times longer than in previous research. The differences suggest that the observed discrepancies in the calculated doses may be attributed to uncertainties in the LT parameter. In the VMAT dose verification, 96.3% of the results were within the tolerance levels (94.8% for relative dose differences and 97.8% for gamma pass rates), with no cases exceeding the action level. These findings demonstrate that the ELM_MS_ meets clinical acceptability; however, further tuning may be necessary to achieve higher accuracy in dose calculations.

In this study, to improve the dose calculation accuracy of the ELM_MS_, LT tuning was initially performed to achieve close agreement between the calculated DZC distributions and film measurements, and subsequently, LG parameters were slightly adjusted to ensure accurate agreement between the calculated and measured point doses in the three VMAT plans. This approach has the advantage of reducing the need for excessive tuning of any single parameter. Several studies have proposed methods for optimizing MLC models that rely solely on adjustments of leaf gap parameters such as the DLG.^1^, ^2^, ^3^, ^4^ Miyasaka et al. reported that a 1‐mm widening of the leaf gap led to approximately a 1.5% increase in the calculated target dose in pelvic VMAT plans.^2^ Middlebrook et al. recommended that the DLG entered in Eclipse be 0.8 mm larger than the measured DLG to reduce the uncertainty of VMAT dose calculation with the M120 MLC model.^3^ Consequently, the agreement between the calculated dose distribution and the film measurement improved, and the pass rate of the gamma index with a criterion of 3%/1.5 mm increased to above 95%. Vieillevigne et al. reported that configuring Eclipse with measured DLG values may lead to significant discrepancies of approximately 5% between measurements and calculations in stereotactic VMAT plans with the HD120 MLC model.^4^ Their investigation found that increasing the measured DLG in Eclipse by 0.7–0.8 mm significantly reduced the discrepancies. Furthermore, they attributed the underestimation of dose calculations for VMAT plans to insufficient modeling of the tongue‐and‐groove (TG) effect in the Eclipse TPS. The TG effect is modeled in the Eclipse TPS by extending the leaf projections in the direction perpendicular to leaf motion; this extension is defined as the TG width parameter. The TG width is not user‐configurable and depends on the MLC model or version of the dose calculation algorithm. According to the latest vendor documentation, the ELM (version 18.0) considers only changes in transmission due to path length inside the leaf body and the dosimetric effect of the rounded leaf tip on the penumbra shape.^7^ In contrast, the current ELM averages inter‐leaf and intra‐leaf transmission during the dose calculation process. Furthermore, this ELM does not model a detailed TG design and adopts a slightly different TG width value compared to the CLM (version 15.5–16.1). For both M120 and HD120 MLCs, the CLM uses a TG width of 0.028 cm, whereas the ELM uses a TG width of 0.033 cm. Incorporating a more detailed inter‐leaf model may further improve the dose calculation accuracy of the ELM without requiring parameter tuning.

As shown in Table 3, the VMAT output variations among TrueBeam linacs are small (<0.6%), indicating the potential feasibility of a unified beam model for each beam energy and MLC type combination. Based on these findings, the ELM_Ave_ was proposed in this study. As shown in Figure 8, the ELM_Ave_ met the tolerance criteria for the relative dose difference and gamma pass rate in all VMAT dose verifications, demonstrating its clinical applicability. The clinical implementation of the ELM_Ave_ can also facilitate a more objective evaluation of user‐defined ELM parameter settings. Note that the leaf positions of the TrueBeam MLC can be individually calibrated by users; therefore, the applicability of the ELM_Ave_ is limited to MLCs with LT and LG values within the ranges presented in Tables 5 and 6. In addition, the use of the ELM_Ave_ in other clinical cases, such as stereotactic VMAT for small brain metastases, has not been comprehensively evaluated. Commissioning that includes smaller target volumes may yield a slightly different optimal ELM_Ave_, which will be investigated in our future studies.

In this study, the calculation uncertainties associated with the open‐beam model based on the RBD, virtual couch model, density scaling of QA devices, and dose calculation algorithm were compensated by tuning the ELM parameters alone. It should be noted that in this study, the ELM parameters were tuned for Acuros XB, and the AAA algorithm was not validated. Consequently, we plan to investigate AAA dose calculations using ELM in our future research. In the dose measurements, phantom setup errors can lead to dosimetric uncertainties, which are estimated to be approximately 0.3%, 0.2%, and 0.7% in Cases #1, #2, and #3, respectively, for a 1‐mm misalignment. The measurements performed using detector array systems such as Delta4 and ArcCHECK may be associated with an uncertainty of at least 1%, even when daily output corrections are applied, due to inter‐institutional variations in detector sensitivity to dose rate and field size. In addition, the detector spacing of 0.5–1.0 cm in these QA systems results in limited spatial resolution of the measured dose profiles. More accurate characterization of measured doses could be achieved using QA devices equipped with high‐density detector arrays. These limitations can affect the determination of the optimal ELM_Ave_.

## 5 CONCLUSIONS

This multi‐institutional study comprehensively evaluated the calculation accuracy and clinical feasibility of the ELM across multiple TrueBeam linacs, with a focus on M120 and HD120 MLCs. According to the results, the ELM_MS_ exhibits acceptable accuracy for VMAT dose calculations across various clinical sites and beam energies. In the ELM, the inter‐leaf structure is represented by a simplified model; therefore, tuning the LT and LG parameters may improve dose calculation accuracy in VMAT plans. The proposed ELM_Ave_ met all clinical tolerance criteria in all verification cases investigated in this study and can serve as a practical solution for efficient commissioning and objective evaluation of ELM parameters across institutions.

## AUTHOR CONTRIBUTIONS

Ryohei Miyasaka conceived of the presented idea. All authors conceived and planned the work that led to the paper. Ryuta Hirai, Yuhi Suda, Ryohei Yamauchi, Norifumi Mizuno, Yuya Suzuki, Mitsunobu Igari, Tomohiro Ohta, Hiroki Nakayama, Yu Arai, Kaito Sakai, Kazushi Hatakeyama, and Hisayuki Miyashita performed the experiment. Masahiko Kurooka and Toru Kawachi discussed the TrueBeam enhanced leaf model uncertainty. Ryusuke Hara supervised the study. Ryohei Miyasaka analyzed the data and all authors discussed the analysis method and results. All authors wrote the paper and approved the final version.

## CONFLICT OF INTEREST STATEMENT

The authors are involved in a collaboration with Varian Medical Systems and financial support was provided.

## Supporting information



Supporting Information
