# Supplementary material for: Multi‐institutional study on the commissioning and clinical implementation of the TrueBeam enhanced leaf model in the Eclipse treatment planning system
Source: J Appl Clin Med Phys. 2025 Nov 25;26(12):e70360. doi: 10.1002/acm2.70360 (PMC12646809; doi:10.1002/acm2.70360)
Supplement: Supplementary file 1 — Supporting Information [file ACM2-26-e70360-s001.docx]

**Supporting Information**

Table S1 Mean calculated dose sensitivity to the Leaf Transmission (LT) parameter in the Dynamic Zebra Crosswalk (DZC) distribution for either the Millennium 120 (M120) or High-Definition 120 (HD120) MLCs.

|  | Mean calculated dose sensitivity to the LT parameter (10^3^ %) | | | | | | | |
| --- | --- | --- | --- | --- | --- | --- | --- | --- |
|  | M120 MLC | | | | HD120 MLC | | | |
|  | 6X | 6X-FFF | 10X | 10X-FFF | 6X | 6X-FFF | 10X | 10X-FFF |
| Low leaf speed region | 1.67 | 1.82 | 1.52 | 1.62 | 1.82 | 1.96 | 1.70 | 1.78 |
| High leaf speed region | 4.39 | 4.96 | 4.04 | 4.45 | 5.24 | 5.88 | 4.94 | 5.43 |

Table S2 Calculated dose sensitivity to the Leaf Gap (LG) parameter in each VMAT plan for either the Millennium 120 (M120) or High-Definition 120 (HD120) MLCs.

| Case # | Calculated dose sensitivity to the LG parameter (%/cm) | | | | | | | |
| --- | --- | --- | --- | --- | --- | --- | --- | --- |
|  | M120 MLC | | | | HD120 MLC | | | |
|  | 6X | 6X-FFF | 10X | 10X-FFF | 6X | 6X-FFF | 10X | 10X-FFF |
| 1 | 1.42 | 1.59 | 1.58 | 1.40 | 1.58 | 1.41 | 1.44 | 1.45 |
| 2 | 1.95 | 1.66 | 2.09 | 1.59 | 1.95 | 1.87 | 2.10 | 1.61 |
| 3 | 3.14 | 3.11 | 3.15 | 3.14 | 3.15 | 2.95 | 2.93 | 3.20 |
| Mean | 2.17 | 2.12 | 2.27 | 2.04 | 2.22 | 2.08 | 2.16 | 2.09 |

Table S3 Summary statistics (mean, standard deviation [SD], and range) of the relative dose differences obtained from VMAT dose verification using machine-specific ELM parameters for either the Millennium 120 (M120) or High-Definition 120 (HD120) MLCs, categorized by X-ray beam energy.

|  | Relative dose difference (%) | | | | | | | |
| --- | --- | --- | --- | --- | --- | --- | --- | --- |
|  | M120 MLC | | | | HD120 MLC | | | |
|  | 6X | 6X-FFF | 10X | 10X-FFF | 6X | 6X-FFF | 10X | 10X-FFF |
| Mean | **−** 1.10 | **−** 1.32 | **−** 1.27 | **−** 1.51 | **−** 0.71 | **−** 0.89 | **−** 0.79 | **−** 1.04 |
| SD | 0.49 | 0.33 | 0.65 | 0.65 | 0.24 | 0.44 | 0.30 | 0.27 |
| Max | **−** 0.33 | **−** 0.51 | **−** 0.24 | **−** 0.68 | **−** 0.34 | **−** 0.15 | **−** 0.24 | **−** 0.73 |
| Min | **−** 2.03 | **−** 1.87 | **−** 2.56 | **−** 2.93 | **−** 1.12 | **−** 1.45 | **−** 1.23 | **−** 1.47 |

Table S4 Summary statistics (mean, standard deviation [SD], and range) of the gamma pass rates (3%/2m, 10%) obtained from VMAT dose verification using machine-specific ELM parameters for either the Millennium 120 (M120) or High-Definition 120 (HD120) MLCs, categorized by X-ray beam energy.

|  | Relative dose difference (%) | | | | | | | |
| --- | --- | --- | --- | --- | --- | --- | --- | --- |
|  | M120 MLC | | | | HD120 MLC | | | |
|  | 6X | 6X-FFF | 10X | 10X-FFF | 6X | 6X-FFF | 10X | 10X-FFF |
| Mean | 99.5 | 99.1 | 99.6 | 99.1 | 99.8 | 99.2 | 99.7 | 99.2 |
| SD | 1.1 | 1.7 | 1.1 | 2.0 | 0.4 | 1.1 | 0.5 | 1.1 |
| Max | 100 | 100 | 100 | 100 | 100 | 100 | 100 | 100 |
| Min | 96.1 | 93.8 | 95.1 | 93.8 | 98.9 | 96.4 | 98.4 | 96.7 |

Table S5 Summary of tuned ELM parameters (LT_Tuned_ and LG_Tuned_) for each TrueBeam equipped with the Millennium 120 MLC.

| TrueBeam # | LT_Tuned_ | | | | LG_Tuned_ (cm) | | | |
| --- | --- | --- | --- | --- | --- | --- | --- | --- |
|  | 6X | 6X-FFF | 10X | 10X-FFF | 6X | 6X-FFF | 10X | 10X-FFF |
| 1 | 0.0181 | 0.0152 | - | - | − 0.0448 | − 0.0270 | - | - |
| 2 | 0.0171 | 0.0166 | 0.0226 | - | − 0.0338 | − 0.0452 | − 0.0397 | - |
| 3 | 0.0213 | 0.0196 | 0.0251 | 0.0234 | − 0.0248 | − 0.0384 | − 0.0213 | − 0.0088 |
| 4 | 0.0191 | 0.0155 | 0.0217 | 0.0184 | − 0.0595 | − 0.0394 | − 0.0489 | − 0.0125 |
| 5 | 0.0192 | 0.0173 | 0.0220 | 0.0201 | − 0.0297 | − 0.0328 | − 0.0189 | − 0.0146 |
| 6 | 0.0205 | 0.0195 | 0.0241 | 0.0231 | − 0.0299 | − 0.0383 | − 0.0264 | − 0.0218 |
| 7 | 0.0182 | 0.0146 | 0.0208 | 0.0168 | − 0.0161 | 0.0005 | − 0.0107 | 0.0302 |
| 8 | 0.0166 | 0.0142 | 0.0223 | 0.0178 | − 0.0070 | − 0.0104 | − 0.0251 | − 0.0034 |
| **Mean** | **0.0188** | **0.0166** | **0.0227** | **0.0199** | **− 0.0307** | **− 0.0289** | **− 0.0273** | **− 0.0051** |
| SD | 0.0016 | 0.0021 | 0.0015 | 0.0028 | 0.0162 | 0.0159 | 0.0129 | 0.0184 |

Table S6 Summary of tuned ELM parameters (LT_Tuned_ and LG_Tuned_) for each TrueBeam equipped with the High-Definition 120 MLC.

| TrueBeam # | LT_Tuned_ | | | | LG_Tuned_ (cm) | | | |
| --- | --- | --- | --- | --- | --- | --- | --- | --- |
|  | 6X | 6X-FFF | 10X | 10X-FFF | 6X | 6X-FFF | 10X | 10X-FFF |
| 9 | 0.0139 | 0.0116 | 0.0160 | 0.0140 | − 0.0131 | − 0.0062 | − 0.0054 | 0.0260 |
| 10 | 0.0169 | 0.0156 | 0.0194 | 0.0184 | − 0.0008 | − 0.0105 | 0.0016 | 0.0056 |
| 11 | 0.0149 | 0.0136 | 0.0181 | 0.0157 | − 0.0114 | − 0.0018 | − 0.0198 | − 0.0043 |
| 12 | 0.0145 | 0.0140 | 0.0189 | 0.0181 | − 0.0255 | − 0.0338 | − 0.0312 | − 0.0331 |
| **Mean** | **0.0151** | **0.0137** | **0.0181** | **0.0165** | **− 0.0127** | **− 0.0131** | **− 0.0137** | **− 0.0014** |
| SD | 0.0013 | 0.0016 | 0.0015 | 0.0021 | 0.0101 | 0.0142 | 0.0147 | 0.0246 |
